# Morphological and Molecular Diversity of Ginger (*Zingiber officinale* Roscoe) Pathogenic Fungi in Chilga District, North Gondar, Ethiopia

**DOI:** 10.3389/ffunb.2021.765737

**Published:** 2022-01-14

**Authors:** Sefinew Tilahun, Marye Alemu, Mesfin Tsegaw, Nega Berhane

**Affiliations:** ^1^Department of Biotechnology, Institute of Biotechnology, University of Gondar, Gondar, Ethiopia; ^2^Department of Agricultural Biotechnology, Institute of Biotechnology, University of Gondar, Gondar, Ethiopia; ^3^Department of Medical Biotechnology, Institute of Biotechnology, University of Gondar, Gondar, Ethiopia

**Keywords:** *Aspergillus*, diversity, pathogenic-fungi, *Penicillium*, pathogen, *Zingiber officinale*

## Abstract

Ginger diseases caused by fungal pathogens have become one of the most serious problems causing reduced production around the world. It has also caused a major problem among farmers in different parts of Ethiopia resulting in a huge decline in rhizome yield. However, the exact causative agents of this disease have not been identified in the state. Although there are few studies related to pathogenic fungus identification, molecular level identification of fungal pathogen was not done in the area. Therefore, this study was undertaken to isolate and characterized the fungal causative agent of ginger disease from the diseased plant and the soil samples collected around the diseased plant from Chilga district, Gondar, Ethiopia. Samples from infected ginger plants and the soil around the infected plant were collected. Culturing and purification of isolates were made using Potato Dextrose Agar supplemented with antibacterial agent chloramphenicol. The morphological characterization was done by structural identification of the isolates under the microscope using lactophenol cotton blue stains. Isolated fungi were cultured and molecular identification was done using an internal transcribed spacer (ITS) of ribosomal DNA (rDNA). A total of 15 fungal morphotypes including 11 *Aspergillus* spp. (73.3%), 2 *Penicillium* spp. (13.3%), and single uncultured fungus clone S23 were isolated from the samples representing all the plant organs and the soil. *Aspergillus* spp. (73.3%) was the most common and seems to be the major causative agent. To the best of our knowledge, this is the first report of ginger pathogenic fungi in Ethiopia identified using ITS rDNA molecular techniques. This study will lay foundation for the development of management strategies for fungal diseases infecting ginger.

## Introduction

Ginger (*Zingiber officinale* Roscoe) (Family: Zingiberaceae) is herbaceous perennial crop plant. It was among the crops that had been introduced and cultivated in Ethiopia in the 13th century (Jansen, 1981; Hegde and Hegde, 2014) and it is currently found growing in wider parts of the country for its fresh and dry rhizomes. It has a significant share in the export commodities of the country with a 0.4% world share (Shimelis, 2021). It was second most widely cultivated spice in Ethiopia next to chilies before it was devastated in 2013 onward due to disease epidemic (Ayenew et al., 2012; Shimelis, 2021). The rhizomes of the crop are used as a spice for cooking as well as alternative medicines (Hailemichael and Tesfaye, 2008). Ginger is propagated vegetatively by planting pieces of rhizome (ca 2.5–5 cm long), on which at least one good bud is present (Jansen, 1981). Despite its uses and contribution to the livelihoods of smallholders and to economy of the country, there has been limited attempt to identify its production challenges in Ethiopia (Geta and Kifle, 2011). Besides, global ginger production drastically reduced from time to time because of different biotic and abiotic factors (Narayanasamy, 2011).

Production and productivity of ginger significantly reduced to a minimum due to various reasons (Zakir et al., 2018; Shimelis, 2021). Various investigations revealed that disease complex, lack of clean planting material, less productivity due to repeated use, and degeneration are the main contributors for low production of Ginger (Senapati and Ghose, 2005). Species of *Aspergillus* and *Penicillium* were reported as the leaf spot and ginger rhizome spoilage diseases (Berza et al., 2012; Meenu and Kaushal, 2017; Mekuria and Alemu, 2020). They were also reported to be the cause of disease in other plants (Photita et al., 2005; Louw and Korsten, 2014; Kazi et al., 2019). The devastating case of ginger bacterial wilt, reported in Ethiopia, was another case of decline in production (Kifelew et al., 2015; Hunduma et al., 2016).

Several fungi species have been reported as the cause of the highest production loss together with other kinds of microbial pathogens at various stages under natural conditions (Robert et al., 2017; Trigiano and Ownley, 2017). Mainly, the destructive and versatile pathogen of *Aspergillus* species and *Penicillium* are common pathogen and most abundant fungus species on the farm and storage rot of ginger (Meenu and Kaushal, 2017). Many investigations showed that ginger diseases are often related to *Pythium* species, *Fusarium oxysporum (F. oxysporum)*, and *Pratylenchus coffeae* (Rahman et al., 2009). There is also a report of an occurrence of ginger bacterial wilt disease, *Ralstonia solanacearum*, in Ethiopia (Hunduma et al., 2016). Berza et al. (2012) identified six genera of fungi from the spoiled ginger samples including *Fusarium, Penicillium, Aspergillus, Rhizopus, Eurotium*, and *Mucor* from southern Ethiopia. According to Rahman et al. (2009), over the last few years, rhizome diseases have affected the crop in many states of India resulting in the decline of rhizome yield and, hence, wilt and soft rot of ginger rhizomes were among the major limiting factors in ginger cultivation.

In the northwestern parts of the country, particularly Chilga district, the livelihood of many poor farmers established on ginger production and it is a major income-generating crop in the area, as a significant quantity of ginger production was from this area [Ethiopian Institute of Agricultural Research (EIAR), 2010]. However, recently, ginger production has become challenging because of unknown disease incidence. Consequently, farmers have lost their additional income from the ginger plantation. Moreover, it is also a national problem that causes the loss of a huge amount of income from foreign currency and the national market. Even though there are studies on the incidence of bacterial wilt disease and rhizome postharvest fungal disease in the southern part of Ethiopia (Berza et al., 2012; Hunduma et al., 2016), disease incidence was not investigated in the other parts of the country such as North Gondar. Moreover, there is no study in Ethiopia that accounts for the identification of fungal disease of ginger using molecular techniques.

It has been difficult to establish appropriate guidelines for the management of ginger production in Ethiopia, mainly due to the lack of substantial knowledge about the main causative agents associated with the ginger disease. Therefore, the identification of fungal pathogens at the species level is very crucial for the necessary intervention mechanisms. Furthermore, for the development of effective management practice, insight into fungal diversity and distribution is needed. Keeping these in view, this study aimed to identify the major pathogenic fungus at the species level using morphological features and DNA sequencing of the internal transcribed spacer (ITS).

## Materials and Methods

### Study Area and Sample Collection

**Figure 1:**
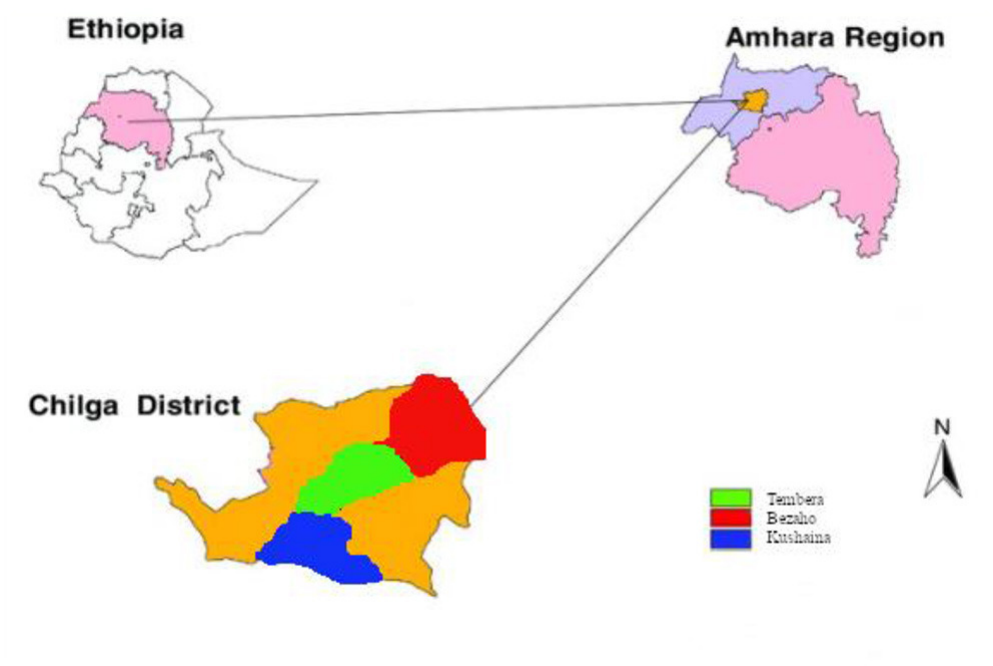
Location of the sampling sites in the Chilga district, northwestern Ethiopia. Inside Chilga district, the red, green, and blue colors represent the three kebeles included in the sampling.

Sampling was conducted in Chilga district, North Gondar Zone, Ethiopia, which is one of the known areas in the cultivation and production of indigenous ginger varieties in Ethiopia. The district had 50 kebeles of which 6 of them were urban kebeles and the rest 44 kebeles were rural kebeles. Elevations in the region range between 731 and 2,383 m above sea level. The district receives about 900 to 1,250 mm annual rainfall (Yonas et al., 2018). The mean minimum temperature and maximum temperature of the district are 19 and 27°C, respectively. From each of the three ginger growing kebeles (Bezaho, Tembera, and Kushayina) of Chilga district, three representative farmlands with severe disease incidence were identified as sampling sites (Figure 1). Samples were collected from August and September 2018 from farmlands. From each field, three different diseased plant materials (leaf, stem, and rhizomes) and a soil sample were collected, for a total of 36 samples, using purposive sampling techniques. Experiments were conducted in the Microbiology and Molecular Laboratories of the University of Gondar.

### Fungal Isolation

Fungal isolates were obtained from the soil and ginger plant samples (leaf, stem, and root) using Potato Dextrose Agar (PDA) (HiMedia Laboratories Pvt. Ltd., Swastik Disha Business Park, Mumbai, India). The medium was autoclaved at 121°C for 15 min and allowed to cool to 50°C before the addition of 0.1 g/l of chloramphenicol. The leave, stem, and rhizome from symptomatic plant part (displayed symptoms) and soil samples were collected and placed in sterile polythene sample bags. Small pieces of the plant sample were placed aseptically in 9 cm diameter petri dishes containing PDA with 0.1 g/l of chloramphenicol and incubated at room temperature (22–25°C) for 5 days. Meanwhile, the healthier part of the stem sample was washed with clean tap water, surface sterilized with 70% ethanol and 2% sodium hypochlorite for 1 min, rinsed in distilled water, and air-dried based with slight modification (Chauhan et al., 2019). Then, the sample was grinded with mortar and pestle in 1 ml distilled water and filtered with 1 mm Whatman filter paper (Ginting et al., 2013). In the meantime, 1 g of soil sample dissolved in 10 ml distilled water and serial dilution was made up to 10^−7^.

A 25-μl of filtrate and 100-μl of aliquots were spread plated on PDA medium. Pure cultures were obtained by transferring the mycelia tips of the culture on 1.5% (w/v) water agar (WA) and allowed to grow overnight. Hyphal tips of the mycelia growth in the WA were later transferred onto PDA supplemented with 0.1 g/l of chloramphenicol.

To identify the distribution and occurrence, isolates from petri dishes of each sample category were recorded. Slant universal bottle was used to preserve the pure cultures of the pathogen and stored within the fridge at 4°C for later use. Appressoria were also produced using slide culture techniques, where 10 mm squares of sterile PDA were placed in an empty petri dish. The edge of the agar was inoculated with spores taken from a sporulating culture and a coverslip was placed over the inoculated agar (Johnston and Jones, 1997). Appresoria formed across the underside of the coverslip were observed for the shape and size of conidia and hyphae septate and the result was recorded.

### Morphological Characterization of the Isolates

To induce conidia production, small pieces of mycelia from the isolates were transferred into 9 cm diameter petri dishes with PDA with 0.1 g/l of chloramphenicol and incubated at 25 ± 1°C for 4 weeks. The isolates were morphologically identified based on cultural and microscopic characteristics using PDA (Kornerup and Wanscher, 1978; Watanabe, 2002; Tafinta et al., 2013). Lactophenol cotton blue mount was used in microscopic identification (Thomas et al., 1991).

### Deoxyribonucleic Acid Extraction and PCR Amplification

Genomic DNA was isolated from all the fungal isolates and was used as a template for PCR. Pure fungi were cultivated in the flasks containing potato dextrose broth at 28°C and 180 rpm for 5 days. The pellets of fungi were collected by centrifugation at 6,000 rpm and the total genomic DNA was extracted using the Gene Elute^TM^ Plant Genomic DNA Purification Kit (Sigma-Aldrich, Burlington, MA) according to the instructions of the manufacturer and the DNA stored at −20°C. The quality of the genomic DNA was checked by nanodrop and gel electrophoresis.

The ITS regions of ribosomal DNA (rDNA), including the 5.8S rDNA, was amplified by using eukaryotic universal primers ITS1 (5′-TCCGTAGGTGAACCTGCGG-3′) and ITS4 (5′-TCCTCCGCTTATTGATATGC-3′) (White et al., 1990). The PCR was carried out in 40 μl reaction volume containing 4 μl (50 ng/μl) of the template DNA, 1 μl each primer [ITS1 (forward) and ITS4 (reverse) with final concentration 0.25 μM/μl from TSINGKE Biological Technology Co., Ltd. (Beijing, China)], 8 μl of 5X FIREPol Master Mix Ready to Load (Solis BioDyne, Riia, Tartu, Estonia, Europe), and 26 μl deionized water. PCR was carried out using TC-04 thermocycler for 30 cycles with an initial denaturation at 95°C for 5 min, cyclic denaturation at 95°C for 30 s, annealing at 60°C for 40 s, and extension at 72°C for 1 min with the final extension of 7 min at 72°C.

**Figure 2:**
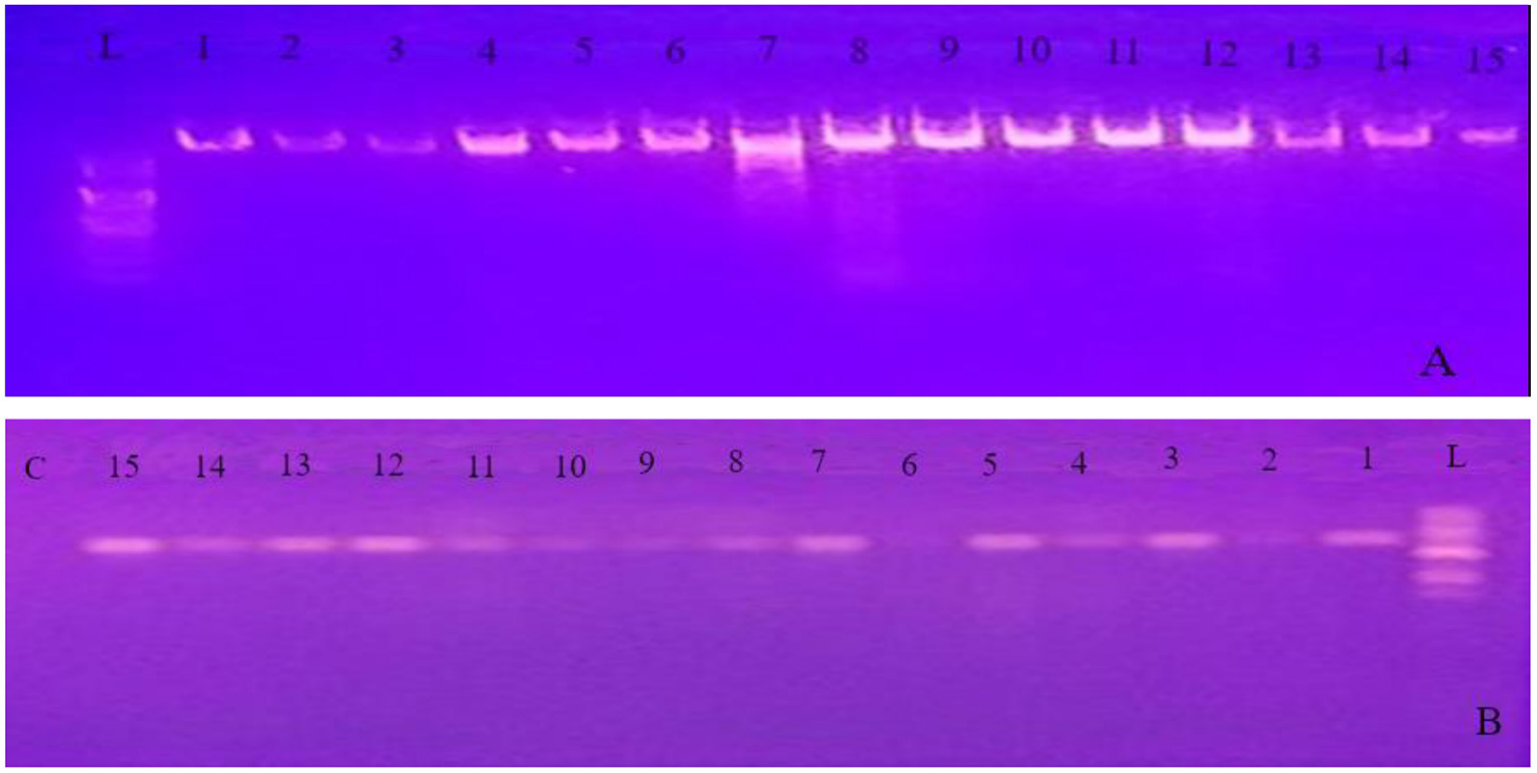
Gel image of genomic DNA **(A)** and PCR product obtained using the internal transcribed spacer (ITS) primer pairs **(B)** for all the 15 isolates. *L* 100 bp ladder; *C* control (PCR mix without primer); 1 up to 15 represents PCR product of the 15 isolates identified in the experiment.

The PCR products were visualized using gel electrophoresis on 1% agarose gel in the 1X Tris-acetate-EDTA (TAE) buffer from Blulux Laboratories Pvt. Ltd., Faridabad, India. (Figure 2). The gels were stained with ethidium bromide and observed in a UV detector (Sambrook et al., 1989). Moreover, approximate molecular sizes of the amplicons were determined using molecular weight marker 100 bp DNA Ladder (HiMedia Laboratories Pvt. Ltd., Swastik Disha Business Park, Mumbai, India). Finally, the PCR products were sent to Macrogen Europe BV Laboratory (Amsterdam, The Netherlands) for sequencing.

### Sequence Analysis and Phylogenetic Tree Construction

The PCR products were sent to Macrogen Europe BV Laboratory (Amsterdam, The Netherlands) for purification and sequencing. Then, the obtained sequence data were compared with other related sequences by Basic Local Alignment Search Tool (BLAST) search using available fungal DNA sequences in GenBank National Center for Biotechnology Information (NCBI) data library (http://www.blast.ncbi.nlm.nih.gov/blast). Multiple sequence alignment and phylogenetic analysis of the rDNA sequences of the isolates obtained in this study were conducted with Molecular Evolutionary Genetics Analysis software version 7.0 (MEGA7) version 7 (Kumar et al., 2016) using the neighbor-joining method with 1,000 bootstrap replicates. The tree stability was evaluated by 1,000 parsimony bootstrap replicates (Felsenstein, 1985).

## Results

### Isolation and Morphological Identification

**Figure 3:**
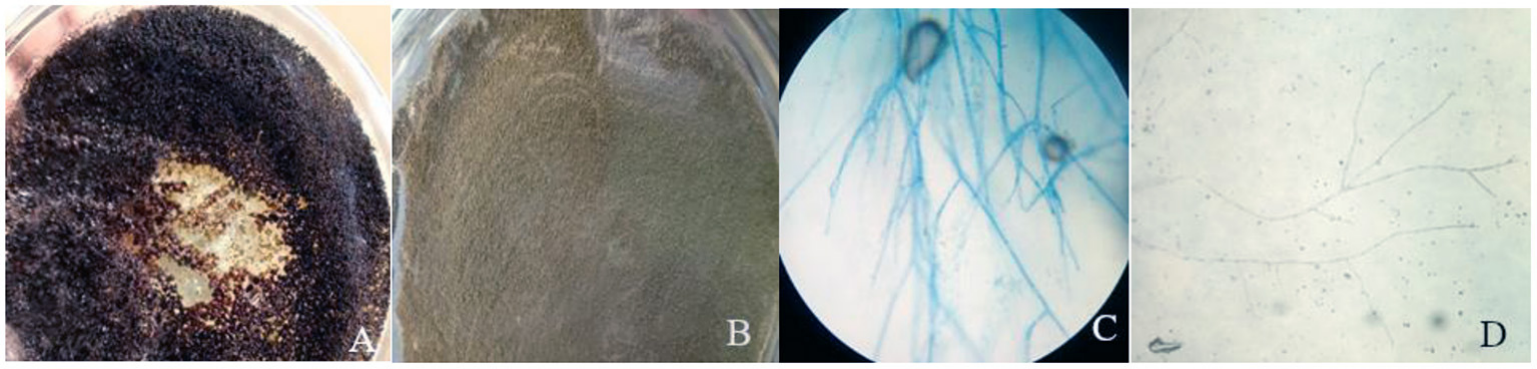
Colony color of plate top of isolate 1 **(A)** and isolate 10 **(B)** and microscopic examination of isolate 10 **(C)** and isolate 5 **(D)**.

A collection of 36 samples was gathered including 28 plant and 9 soil samples. Using morphological characteristics, 15 different non-overlapping fungal colonies were identified using the pure culture technique (Kornerup and Wanscher, 1978; Watanabe, 2002; Tafinta et al., 2013). The species identification was first carried out according to colony or hyphae morphology of the fungal cultures and spores (Figure 3). Initially, some of the fungal isolates were identified to genus level. Among them, *Aspergillus* and *Penicillium* were common. Later on, using the data of ITS1 region of rDNA sequences analysis together with morphological characteristics (Table 1), 7 species in two genera, *Aspergillus* and *Penicillium*, were identified.

**Table 1:** Cultural characteristics of fungal isolates obtained from different plant parts and soil samples.

**Isolate code**	**Spore characteristics**			**Fungus**
	**Colony shape and color**	**Conidiophore shape**	**Conidial shape**	
Isolate 1	Brown or black,	Swollen heads, smooth, hyaline,	Circular	*A. niger*
Isolate 2	Yellow white	Swollen heads	Olive to brown	*A. tamarii*
Isolate 3	Brownish black	Swollen heads, smooth, hyaline,	Circular	*A. niger*
Isolate 4	Gray/whitish	Stick like		Uncultured fungus clone S23
Isolate 5	Hyaline Oily and green	Branched, form sporulating swollen head	Long-ovoid to cylindrical, compacted into columns	*A. flavus*
Isolate 6	Thick/compact, yellowish-green	Conidia in dry heads		*A. oryzae*
Isolate 7	Yellowish-green	Phialides upright, short, highly branched and brushlike	Rounded, closely packed, disordered chains.	*P. griseofulvum*
Isolate 8	Conidia in dry heads, green-white	Swollen heads	Uniseriate conidial head and a rough conidia	*A. flavus*
Isolate 9	Green with a white circle	Swollen heads, swollen apically, phialides	Tiny spherical to subspherical, in chain	*A. flavus*
Isolate 10	Whitish to brown	Highly branched, brush-like	Spherical to subspheroidal	-
Isolate 11	Yellow-green to white	Swollen heads, swollen apically, phialides	Spherical to subspherical,	*A. flavus*
Isolate 12	Yellow-green to white	Swollen heads, swollen apically, phialides	Spherical to subspherical,	*A. flavus*
Isolate 13	Conidia in dry heads, green-white	Rounded heads	Uniseriate conidial head and a rough conidia	*A. nomius*
Isolate 14	Brownish White	Phialides upright, branched and brushlike	Spherical to subspheroidal,	*P. crustosum*
Isolate 15	Compact, yellowish-green	Rounded heads	Circular	*A. oryzae*

The color of the colony was used as the first level of identification. From the observations made, mold fungal growths were seen from samples of both the infected ginger plant part inoculums and soil sample culture plates. Among them, five colonies were greenish with white circle; two yellow-green other one yellow and white-shaded colonies and two black colonies were obtained. From microscopic observation, all these colonies were characterized by having colorless mycelium with swollen heads, which are believed to be members of the genera *Aspergillus*.

The other group of isolates (included four isolates) was characterized by having long branched brush-like mycelium with different colony colors (Table 1), identified as the genera *Penicillium*. The first level of identification showed that all the isolates were belong to the genus *Aspergillus* and *Penicillium*, indicated that they were major causes of ginger disease in the area. From the microscopic characteristics, those that could be referred to as a well-defined species were *A. niger, A. tamarii, A. flavus, A. oryzae*, and *A. nomius*. Further identification to species level was conducted using ITS1-5.8S-ITS4-based sequence comparison using DNA-DNA BLAST. Hence, based on morphological and molecular characteristics, 15 isolates of fungi were determined from this study, which belongs to 10 isolates of *Aspergillus* which was presented by 5 species; *A. niger (2), A. tamarii (1), A. flavus (5), A. oryzae (2), A. nomius (1)* and 2 species of *Penicillium*; a *Penicillium griseofulvum (P. griseofulvum)* and a *Penicillium crustosum (P. crustosum)* one uncultured fungus and one unknown isolate.

### Percentage Incidence of Fungal Genera Among Plant Organs and the Soil

The distribution of isolates does not show any clear pattern among the sample types. The number of isolates and their distribution in each plant organ studied and the soil is given in Table 2. The smallest number of the isolates was observed in the soil sample (occupied by 6 isolates) and the largest number of isolates was observed in the root or rhizomes (occupied by 9 isolates). *A. flavus* was isolated from all the sample types.

**Table 2:** Frequency of occurrence of the different fungi species, as isolated from the plant parts and the soil, on Potato Dextrose Agar (PDA) containing chloramphenicol (0.1 g/l).

**Fungus**	**Isolate code**	**Frequency**	**Sample type**			
			**Leaf**	**Stem**	**Rhizome**	**Soil**
*A. niger*	Isolate 1	3	+	+	+	-
*A. tamarii*	Isolate 2	2	+	-	+	-
*A. niger*	Isolate 3	3	+	-	+	+
Uncultured fungus clone S23	Isolate 4	2	-	+	+	-
*A. flavus*	Isolate 5	4	+	+	+	+
*A. oryzae*	Isolate 6	1	-	+	-	-
*P. griseofulvum*	Isolate 7	3	+	+	+	-
*A. flavus*	Isolate 8	1	+	-	-	-
*A. flavus*	Isolate 9	1	-	-	-	+
——–	Isolate 10	2	-	+	-	+
*A. flavus*	Isolate 11	1	+	-	-	-
*A. flavus*	Isolate 12	2	-	-	+	+
*A. nomius*	Isolate 13	1	+	-	-	-
*P. crustosum*	Isolate 14	3	-	+	+	+
*A. oryzae*	Isolate 15	1	-	-	+	-
Total		30	8	7	9	6

*+, the presence of corresponding fungal isolate in the sample; -, absence of corresponding fungal isolate in the sample*.

**Figure 4:**
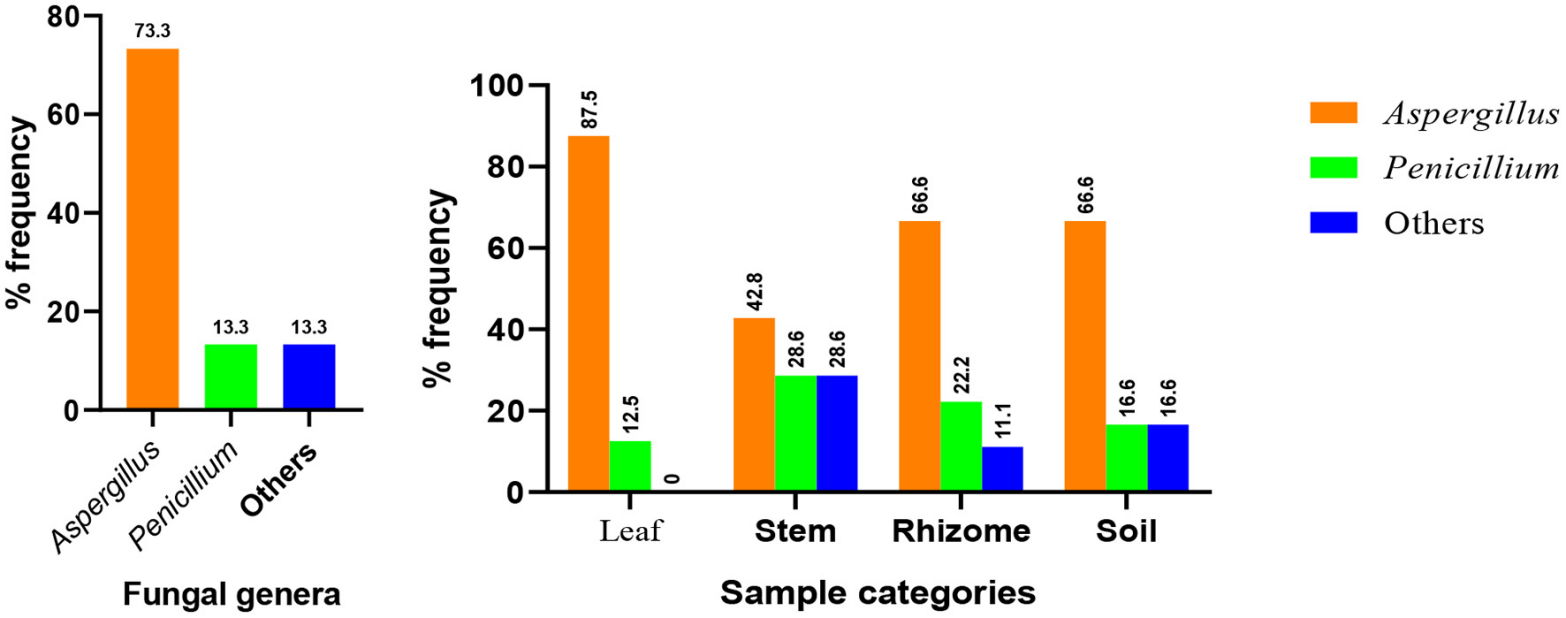
Distribution of major fungal genera identified from the whole samples (left) and each sample types (right).

Based on data shown in Table 2 and Figure 4, all the plant organs of ginger and the soil were inhabited by pathogenic fungus. Percentage incidence of fungi isolated from different samples varies among leaf, stem, rhizome, and soil samples (Figure 4, right). The percentage incidence of fungi from diseased ginger leaves ranged from 12.5 (*Penicillium*) to 87.5% (*Aspergillus*). In rhizome samples, 66.7% of incidence was due to *Aspergillus* followed by *Penicillium* (28.5%) and others fungal genera (11.1%). In a similar manner, incidence of fungus from soil samples collected around the diseased plant was dominated by *Aspergillus* (66.7%) followed by *Penicillium* and other fungal genera. In the cases of stem samples, all the two genera and other isolates have nearly similar percentage incidence. Generally, 73.3% of the isolate identified as *Aspergillus* species (Figure 4, left), indicated that it is the most important ginger disease-causing genera in the area. *Penicillium*, an uncultured fungus clone and an isolate without a significant match in the NCBI, was also found to be responsible for fungal disease in the area.

### Deoxyribonucleic Acid Sequence Analysis

Molecular methods have dramatically increased our knowledge of fungi by helping to resolve relationships within the genus (Blackwell, 2011). To support the morphological data and further identification to species level, study was conducted using sequence analysis of parts of rDNA. The ITS1/ITS4 primer pair was used to amplify the intervening 5.8S rDNA and the adjacent ITS1 and ITS2 regions. Quality of both genomic DNA and PCR products was confirmed by agarose gel electrophoresis (Figure 2). The approximate molecular sizes of the PCR products were 570 bp. Similar amplicon sizes of the PCR products using ITS1/ITS4 primer pairs were reported (Henry et al., 2000; Bhadury et al., 2011; Demirel et al., 2013; Zarrin et al., 2016). The nucleotide sequences received from Macrogen Europe BV Laboratory (Amsterdam, The Netherlands) were aligned through the NCBI Basic Local Alignment Search Tool for nucleotide sequence (BLASTN) search engine and the most appropriate similar sequence was selected from the GenBank based on expected value and percent identity.

Based on BLAST search, the similarity of the isolated fungi to the closest species available in the NCBI varied from 82 to 99% (Table 3). Out of the 15 isolates, 14 of them were identified into 7 species and 1 uncultured fungus with a significant match. However, isolate 10 identified from stem and soil samples could not have a significant match under the NCBI fungal database. Unless we mask low complexity sequences, there is no significant similarity found for isolate 10. The match we found, when we mask low complexity sequences, is not fungus species. Based on the blast search of rDNA sequences, 11 isolates from different sources were identified as *Aspergillus* species. Blast search results of sequences of isolates 7 and 14 were showed 98 and 99% similarity with *P. griseofulvum* and *P. crustosum*, respectively. This study also identified isolate 4 as uncultured fungus clone S23 with 100% similarity.

**Table 3:** Basic alignment search result of the 18S ribosomal DNA (18S rDNA) sequence of the 15 isolates.

**Number of isolates**	**NCBI accession number**	**Closest NCBI database match**	**Percentage identity**	***E*-value**
Isolate 1	MT609916	*A. niger*	91	7e^−105^
Isolate 2	KR149638	*A. tamarii*	100	0.0
Isolate 3	MW958029	*A. niger*	91	0.0
Isolate 4	KY978255.1	Uncultured fungus clone S23	100	0.001
Isolate 5	MT635198.1	*A. flavus*	99	0.0
Isolate 6	MG786521.1	*A. oryzae*	89	6e^−80^
Isolate 7	KJ191338.1	*P. griseofulvum*	98	0.0
Isolate 8	MN420994	*A. flavus*	93	8e^−87^
Isolate 9	KY234263.1	*A. flavus*	82	4e^−40^
Isolate 11	MN533852.1	*A. flavus*	88	2e^−115^
Isolate 12	MK645222.1	*A. flavus*	99	0.0
Isolate 13	LN482574	*A nomius*	100	0.0
Isolate 14	MW538312.1	*P. crustosum*	99	0.0
Isolate 15	LN482587.1	*A. oryzae*	96	0.0

Generally, *Aspergillus* species, dominantly *A. flavus*, was most frequent during the isolation process from all the leaf, root, and soil samples. Molecular analysis indicated that isolates having different morphology were not always belongs to different species, as implied by the fact that isolate 5, isolate 8, isolate 9, isolate 11, and isolate 12, which have a slight differences in morphological characteristics, are members of single species, *A. flavus*. Similar even was shown between the morphotypes of isolates 1 and 3, isolates 6 and 15, and isolates 8 and 15 (Table 1).

### Phylogenetic Analysis and Evolutionary Relationship Between Isolates

**Figure 5:**
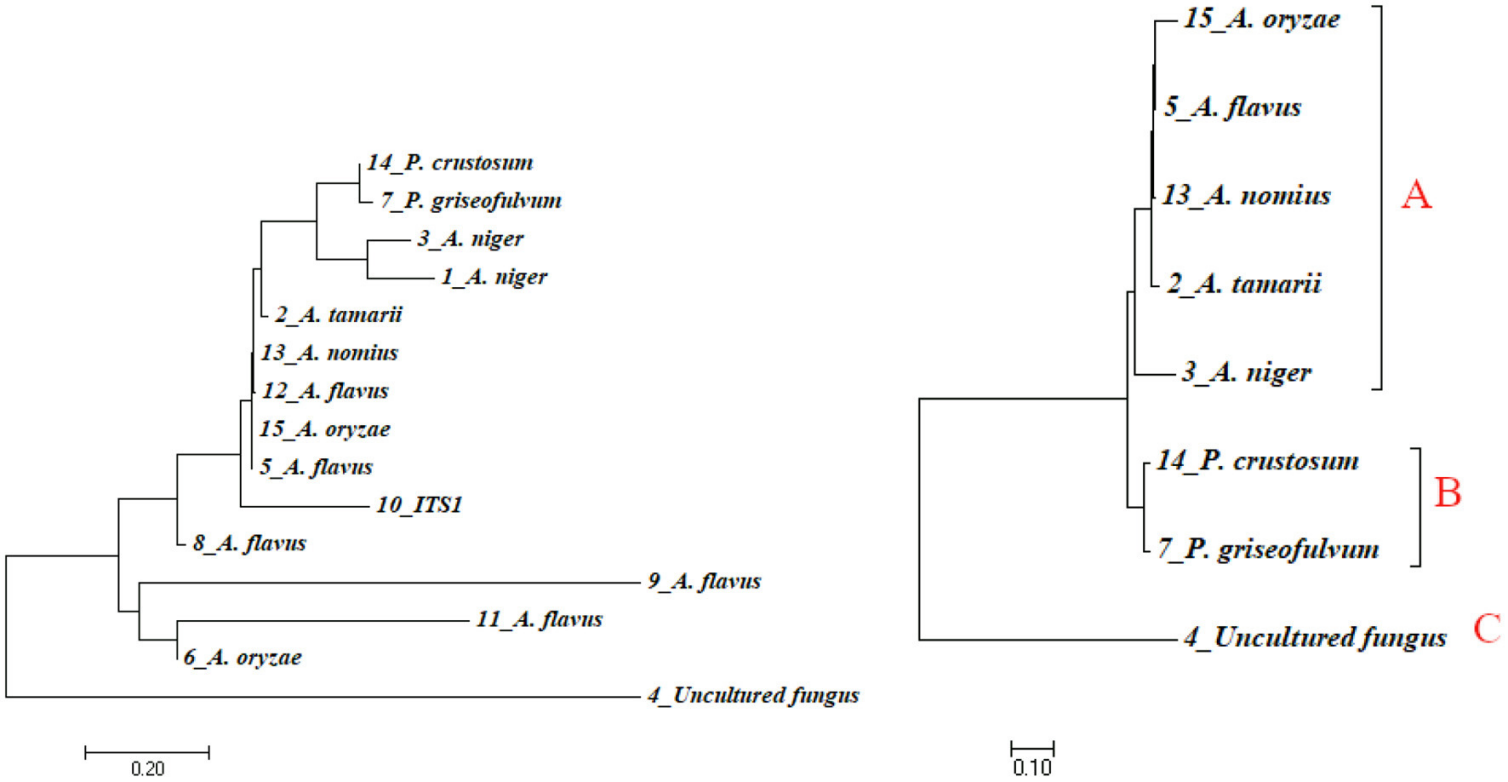
Phylogenetic tree indicating evolutionary relationships of taxa based on neighbor-joining method (left) and maximum likelihood method (right) using sequence data of isolates. Numbers above branches are bootstrap values 1,000.

Phylogenetic approaches based on the neighbor-joining method (Figure 5, left) were undertaken to get a clear understanding of the grouping of the fungal sequences generated from this study with published 18S rDNA sequences. FASTA sequence was used for multiple alignment and phylogenetic tree construction using MEGA7 version 7. Species such as *A. flavus* were found in a different cluster in the tree. This fact, together with the presence of slight morphological variation between them, illustrated the fact that those morphotypes could be different strains of *A. flavus*. To find out a clearer relationship between species, we developed a phylogenetic tree using only representatives from each species (Figure 5, right). In the case that there is more than one representative in a species, we take the isolate with the highest percentage identity. Cluster analysis based on eight representative isolates reveals the presence of three major clusters denoted as A, B, and C (Figure 5, right). Cluster A consists of five species of the general *Aspergillus*. Cluster B consists of two *Penicillium* species (*P. crustosum* and *P. griseofulvum*). Cluster C has one unnamed isolate identified as uncultured fungus clone S23. Uncultured fungus clone S23 is not involved in any cluster in both the trees indicated that it is relatively divergent from the other taxa identified in this study.

In the phylogenetic tree, the representative isolates of *Aspergillus* were highly clustered within the clades comprising their close relative isolates. All the five *Aspergillus* isolates were clustered in one clade. Similarly, the two species of *Penicillium* were clustered in the same clade. Uncultured fungus clone S23 was clustered in its own clade indicated that uncultured fungus clone S23 is closely related to neither *Penicillium* nor *Aspergillus* species.

## Discussion

A large number of fungal species have been reported worldwide to cause disease in ginger. These fungi belong to a different genus including *Aspergillus, Penicillium, Pythium, Fusarium*, and *Phyllosticta* (Dake, 1995; Pawar et al., 2008; Moreira et al., 2013; Meenu and Kaushal, 2017). In this study, several species of the genus *Aspergillus* and *Penicillium* were isolated and identified from diseased ginger samples (leaf, stem, and rhizomes) and the soil samples around the diseased plant.

In this study, colony and spore characteristics were used to characterize fungal isolates from diseased plant organs and the soil. The ITS rDNA fragments of isolates were amplified and their PCR amplicons were sequenced and aligned with other published sequences in the NCBI to identify the species using sequence similarity and maximum identity.

Colony and spore characteristics have been useful taxonomic characteristics for species differentiation in fungus (Johnston and Jones, 1997; Photita et al., 2005). However, some characteristics such as colony color and conidiophore structure for various species overlap. Moreover, members of the same species may have different morphological characteristics such as conidial shape and colony color. In this study, isolate 9, isolate 11, and isolate 12 differed in morphological characteristics; however, the DNA sequence analysis indicated that they belonged to the same species, namely, *A. flavus*. Therefore, it is obvious that the colony and spore characteristics alone do not precisely distinguish between species. Generally, it was possible to distinguish between individual isolates of *Aspergillus* and *Penicillium* from the colony and spore morphology, although not between species of the same genera.

Morphology-based taxonomy sometimes may not resolve species accurately due to overlapping characters and a high degree of phenotypic plasticity (Senanayake et al., 2020). Besides, amplification of ITS2/5.8S rDNA and molecular typing shows potential as a rapid technique for identification of fungi (Ferrer et al., 2001). Using the universal fungal primers (ITS1/ITS4), the PCR products of about 570 bp were obtained from all of the species (Figure 2). Based on the BLAST search of ITS1-ITS4 region of rDNA sequences, 11 isolates from different sources were shown 82–99% similarities with *Aspergillus* species. Light-microscope examination of the isolates spore characteristics (Table 1) supported this result that all the 11 isolates notably distinguished as having swollen-headed conidiophore, which was a typical feature to genus *Aspergillus*. All the rest four isolates, which we initially thought to be placed in the genus *Penicillium* due to their brush-like conidiophore with different sizes, were identified as *Penicillium* (two of the isolates), uncultured fungus and one unidentified isolate through DNA sequence analysis.

The highest matches for isolate 4 were to an uncultured fungus clone S23 18S rRNA clone (Accession No. KY978255.1) amplified from the sample collected from a tropical hot and humid climate in the South-Eastern coast of India. This GenBank sequence is, perhaps, closely related members of the Ascomycota fungus (Valsan et al., 2015) whereas the phylogenetic tree indicated that uncultured fungus clone S23 is not involved in any cluster indicated that it is relatively divergent from the genus identified in this study. To help reduce the noise and get clearer information, a phylogenetic tree was developed using only representative species (Figure 5, right). Uncultured fungus clone S23 was still clustered in a separate and its own clade may indicate that uncultured fungus clone S23 is closely related neither to *Penicillium* nor *Aspergillus* species. DNA sequencing technologies believed to enhance the accuracy of fungal crop disease detection (Jain et al., 2019). Hence, further identification of this isolate may help to know whether it is a new species or not. In this grouping, the representative isolates of the genus *Aspergillus* were highly clustered within the clades comprising their close relatives. All the five *Aspergillus* species were clustered in one clade. Still, the morphological data imply otherwise as illustrated by the difference in the structure of the conidiophore between them. Generally, BLAST search indicated that ginger fungal sequences amplified in this study broadly represent taxa within the genera *Aspergillus* and *Penicillium* and one morphotypes showed robust groupings with known taxa in the published data in the NCBI.

*Fusarium oxysporum, F. solani, F. zingiber*, and *Pythium graminicolum* were among the most common fungi responsible for the disease of ginger causing a drastic reduction in crop yield in different countries (Trujillo, 1964; Senapati and Ghose, 2005; Rosangkima et al., 2018). In this study, one of the most abundant fungal genera responsible for disease in ginger was *Aspergillus*, which accounts for 73.3% of the whole incidence. Out of the 11 isolates identified in this genus, 5 of them were *A. flavus* (Figure 4, left). A similar report from food grain crops was reported by Thilagam et al. (2016) in which the predominating fungal species were found to be *A. flavus* (63%) followed by *A. niger* (16%). Similarly, Toma and Abdulla (2013) has identified *A. flavus* and *A. tamarii* from black pepper, *A. niger* and *Penicillium* from red tea, and *A. flavus* and *Penicillium* from garlic. *A. niger* and *A. flavus* were the most prevalent species isolated from sixteen spice and medicinal plants. Okayo et al. (2020) also reported the predominant occurrence of toxigenic *A. flavus* from groundnut kernels in Kenya. In their study on post-harvest spoilage of ginger in Ethiopia, Berza et al. (2012) has identified *Aspergillus* as a one of the predominant species together with Fusarium and *Pencillium*. Al-hindi et al. (2011) have isolated and identified *A. tubingensis* from peach and *A. niger* from apple. Raper and Fennell (1965) indicated that *Aspergillus* section Flavi was the most predominant with 57% followed by section Nigri with 27% from maize and 58% of section Flavi followed by 26% of section Nigri from the soil. In a more related study, Pawar et al. (2008) have been reported that *A. niger* is a pathogen of *Zingiber officinale*.

The second most important and predominant genera identified in this study was *Penicillium*. This result is in agreement with the finding of Demirel et al. (2013), who reported *P. griseofulvum* and *P. crustosum* as important pathogens of mature fruits and cereal grains. In this study, *P. aurantiogriseum* and *P. griseofulvum* were found to be the actual causative agents for the blue mold disease of apple. *P. crustosum* was also identified as an apple pathogenic fungal species by Louw and Korsten (2014). According to this study, *P. crustosum* was reported as the second most aggressive pathogen next to *P. expansum* in different types of apple cultivars. Moreira et al. (2013) identified *P. commune* as one of the pathogens in postharvest rot of ginger rhizomes in the Serrana region of Espírito Santo, Brazil. Despite the few reports on the mycotoxins and postharvest rot in ginger rhizomes by some species of *Penicillium* (Moreira et al., 2013; Mamo et al., 2020), there is no study reporting pathogenicity of *P. crustosum* as well as *P. griseofulvum* on ginger. This study is the first study to report these two strains of *Penicillium* as a causative agent in ginger.

Reports indicated that the highest incidence of pathogenic fungus is usually observed in rhizomes and soil (Senapati and Ghose, 2005; Moreira et al., 2013; Meenu and Kaushal, 2017). Similarly, this study finds out that the largest number of isolates was observed as root/rhizomes (occupied by 9 isolates). In all the ginger plant samples and the soil, the most prevalent pathogen was *Aspergillus*. For example, in rhizomes, more than 66% of these isolates were among species of the genus *Aspergillus*.

Although, studies indicated that *Fusarium* spp., *Rosellinia* spp., *and Pythium* spp. were the most common species causing rhizome soft rot disease around the world (Dake, 1995; Stirling, 2002; Kavitha and Thomas, 2008; Rosangkima et al., 2018); other genera of fungi such as *Penicillium, Aspergillus, Mucor, Eurotium*, and *Rhizopus* have also been isolated from ginger as causal agents of rhizome rot (Berza et al., 2012; Mekuria and Alemu, 2020). In agreement with this, several species of *Penicillium* and *Aspergillus* were identified as the prevalent causative agents of ginger disease in this study in Ethiopia. Uncultured fungus clone and an isolate without a significant match in the NCBI were also found to be the causative agent of ginger disease in the area. Although the genome diversity of fungal species is studied with increasing intensity, the vast majority of fungal species remain unknown (Blackwell, 2011; Schoch et al., 2012). Hence, it is important to make further study on the two unknown isolates to confirm whether they are new/novel strains or not.

Uncultured fungus clone S23 was not reported before this time as a causative agent for the ginger disease. Therefore, our detection in this study may be the first report as a causative agent in ginger disease. However, further investigation on its pathogenicity may be required.

The phylogenetic tree from this study shows three loose outgroups of fungal isolates; the first one comprises the five representative isolates of *Aspergillus* species. The second outgroup contains two species of *Penicillium* and an uncultured fungal strain represents the third outgroup. The rDNA genes, generally used in the taxonomic and identification studies (White et al., 1990; Glass and Donaldson, 1995), were confirmed in this study. The two approaches used in this study—morphological and molecular identification—were together able to distinguish between isolates and characterize individual isolates from diseased fungal organs and the soil.

## Conclusion

The findings of this study indicated the diversity occurrence and distribution of ginger pathogenic fungi around Chilga district, Gondar, Ethiopia. To the best of our knowledge, this is the first time that molecular methods were used to identify fungal pathogens of ginger in North Gondar, Ethiopia. The isolated fungi from the diseased ginger samples and the soil around the diseased plant in Chilga, Gondar were identified as *Aspergillus* (11 spp.), *Penicillium* (2 spp.), and an uncultured fungal strain. Of the several species isolated from the diseased ginger and soil samples, members of *Aspergillus*, especially *A. flavus*, were the most prevalent pathogenic fungi across the fields of farmers.

Our identification of fungi causing ginger disease could be the first step toward further studies to develop an integrated crop management program to prevent ginger pathogen in Chilga district and other regions of Ethiopia. It is suggested that further investigation is required to study the diversity of ginger pathogenic fungi throughout ginger-producing areas of the country, which could help to design proper, effective, and ecologically safe management strategies, particularly in Ethiopia and other parts of the world, in general. Performing pathogenicity test for isolates in the future believed to be valuable in identifying the degree of pathogenicity.

In addition to the morphology-based identification, sequence analysis from the ITS regions has helped us progress toward a better understanding of the taxonomy of isolates, but further molecular analysis using other genes and more sensitive approaches is required to identify some of the species such as uncultured fungus clone S23. Moreover, further investigation has to be done to check for the possible pathogenic microorganism such as bacteria, viruses, and nematodes as well as the environmental factor related to the enhancement of the opportunistic fungal disease. This study was only able to isolate fungal species from a restricted area of the country. Further study is needed to fully investigate the diversity of economically important ginger pathogenic fungi in Ethiopia. This study will lay the foundation for the development of management strategies for fungal diseases infecting ginger.

## Data Availability Statement

The DNA sequence data presented in this study were deposited in the NCBI repository with accession numbers: OL868953–OL868966.

## Author Contributions

ST took part in designing the study, data collection, and writing of the manuscript. MT designed the study and took part in data collection and analysis. MA took part in data collection and writing. NB took part in review and editing the manuscript. All authors contributed to the article and approved the submitted version.

## Conflict of Interest

The authors declare that the research was conducted in the absence of any commercial or financial relationships that could be construed as a potential conflict of interest.

## Publisher's Note

All claims expressed in this article are solely those of the authors and do not necessarily represent those of their affiliated organizations, or those of the publisher, the editors and the reviewers. Any product that may be evaluated in this article, or claim that may be made by its manufacturer, is not guaranteed or endorsed by the publisher.
